# Investigating the Central Nervous System Disposition of Actinomycin D: Implementation and Evaluation of Cerebral Microdialysis and Brain Tissue Measurements Supported by UPLC-MS/MS Quantification

**DOI:** 10.3390/pharmaceutics13091498

**Published:** 2021-09-17

**Authors:** Julia Benzel, Gzona Bajraktari-Sylejmani, Philipp Uhl, Abigail Davis, Sreenath Nair, Stefan M. Pfister, Walter E. Haefeli, Johanna Weiss, Jürgen Burhenne, Kristian W. Pajtler, Max Sauter

**Affiliations:** 1 Hopp Children’s Cancer Center Heidelberg (KiTZ), 69120 Heidelberg, Germany; j.benzel@kitz-heidelberg.de (J.B.); s.pfister@kitz-heidelberg.de (S.M.P.); k.pajtler@kitz-heidelberg.de (K.W.P.); 2 Division of Pediatric Neurooncology, German Cancer Consortium (DKTK), German Cancer Research Center (DKFZ), Im Neuenheimer Feld 580, 69120 Heidelberg, Germany; 3 Faculty of Biosciences, Heidelberg University, Im Neuenheimer Feld 234, 69120 Heidelberg, Germany; 4 Department of Clinical Pharmacology and Pharmacoepidemiology, Heidelberg University Hospital, Im Neuenheimer Feld 410, 69120 Heidelberg, Germany; gzona.bajraktari-sylejmani@med.uni-heidelberg.de (G.B.-S.); philipp1.uhl@med.uni-heidelberg.de (P.U.); walter-emil.haefeli@med.uni-heidelberg.de (W.E.H.); johanna.weiss@med.uni-heidelberg.de (J.W.); juergen.burhenne@med.uni-heidelberg.de (J.B.); 5 Department of Pharmaceutical Sciences, St. Jude Children’s Research Hospital, 262 Danny Thomas Place, Memphis, TN 28105, USA; Abigail.davis@stjude.org (A.D.); sreenath.nair@stjude.org (S.N.); 6 Department of Pediatric Hematology, Oncology and Immunology, Heidelberg University Hospital, Im Neuenheimer Feld 430, 69120 Heidelberg, Germany

**Keywords:** actinomycin D, UPLC-MS/MS, central nervous system, micro-sampling, cerebral microdialysis

## Abstract

Actinomycin D is a potent cytotoxic drug against pediatric (and other) tumors that is thought to barely cross the blood–brain barrier. To evaluate its potential applicability for the treatment of patients with central nervous system (CNS) tumors, we established a cerebral microdialysis model in freely moving mice and investigated its CNS disposition by quantifying actinomycin D in cerebral microdialysate, brain tissue homogenate, and plasma. For this purpose, we developed and validated an ultraperformance liquid chromatography–tandem mass spectrometry assay suitable for ultra-sensitive quantification of actinomycin D in the pertinent biological matrices in micro-samples of only 20 µL, with a lower limit of quantification of 0.05 ng/mL. In parallel, we confirmed actinomycin D as a substrate of P-glycoprotein (P-gp) in in vitro experiments. Two hours after intravenous administration of 0.5 mg/kg, actinomycin D reached total brain tissue concentrations of 4.1 ± 0.7 ng/g corresponding to a brain-to-plasma ratio of 0.18 ± 0.03, while it was not detectable in intracerebral microdialysate. This tissue concentration exceeds the concentrations of actinomycin D that have been shown to be effective in in vitro experiments. Elimination of the drug from brain tissue was substantially slower than from plasma, as shown in a brain-to-plasma ratio of approximately 0.53 after 22 h. Because actinomycin D reached potentially effective concentrations in brain tissue in our experiments, the drug should be further investigated as a therapeutic agent in potentially susceptible CNS malignancies, such as ependymoma.

## 1. Introduction

Actinomycin D is a cytostatic antibiotic drug that is effective in the treatment of gestational trophoblastic neoplasia, Wilms tumor, rhabdomyosarcoma, and Ewing’s sarcoma, and is being evaluated for NPM1-mutated acute myeloid leukemia [1,2]. It is assumed to act via insertion (i.e., intercalation) into the DNA helix [3,4], thus inhibiting DNA transcription and RNA synthesis [5], and subsequently preventing protein synthesis. Furthermore, actinomycin D also stabilizes cleavable DNA complexes of topoisomerase I and II, thus facilitating the formation of single-strand DNA breaks [6,7]. Although applied for decades, few studies investigated the pharmacokinetics of actinomycin D [8,9,10,11,12]. Actinomycin D is reported as a substrate of P-glycoprotein (P-gp) [13], one of the most important efflux transporters of the blood–brain barrier (BBB), which can limit the penetration into the brain of a vast spectrum of substrates [14]. Due to low cerebrospinal fluid concentrations and its substrate characteristics for P-gp, it is not expected to cross the BBB to any relevant extent [10]. Nevertheless, comprehensive information on its central nervous system (CNS) disposition is lacking. However, previous studies suggested a potential role of actinomycin D in the treatment of pediatric CNS neoplasms: Preclinical studies revealed efficacy in in vitro models of ependymoma [15] and, to a certain degree, in orthotopic mouse models of glioblastoma, medulloblastoma, and embryonal tumors with multilayered rosettes (ETMR) [16,17,18]. Therefore, our aim was to establish a suitable experimental setup to accurately determine the still unclear CNS disposition of actinomycin D. This setup combined quantification of actinomycin D by ultraperformance liquid chromatography–tandem mass spectrometry (UPLC-MS/MS) in cerebral microdialysis microsamples from freely moving mice with measurements in brain tissue and plasma.

**Figure 1:**
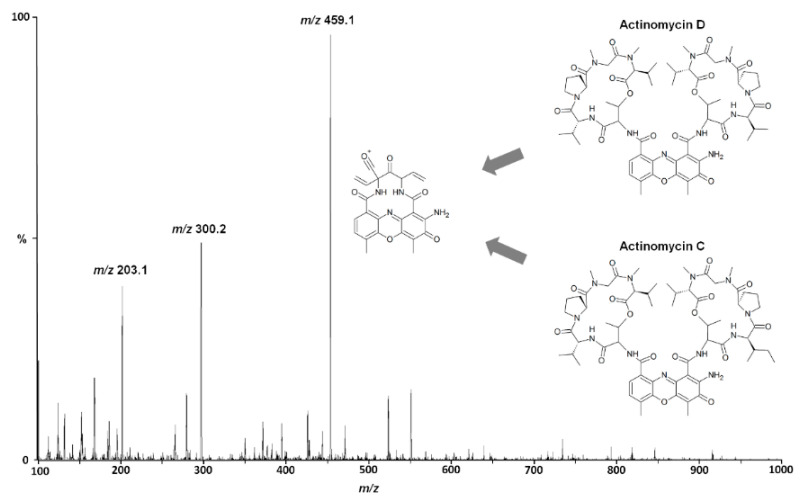
Positive product spectrum (MS/MS) of the [M+H]^+^ signal (m/z 1255.6) of actinomycin D in collision-induced decomposition using a collision energy of 62 V. The structures of actinomycin D, actinomycin C, and their monitored common product ion (*m/z* 459.1) are additionally depicted. For details on the product ions and fragmentation scheme of actinomycin D, the reader is referred to the work of Thomas and co-workers [29].

Actinomycin D consists of two identical pentapeptide lactone rings (cyclodepsipeptides; sequence: L-threonine, D-valine, L-proline, sarcosine, and D-methylvaline) cyclized via the hydroxyl group of the side chain of threonine and the carboxyl function of D-methylvaline; these are additionally connected via amide bonds of the amino function of the threonines (peptide N-termini) to the carboxyl groups of 2-amino-4,6-dimethyl-3-oxo-3H-phenoxazine-1,9-dicarboxylic acid (Figure 1). Due to the cyclic form of the depsipeptides and the stability of the phenoxazine system, actinomycin D exhibits collision-induced dissociation (CID) mass transitions with a low intensity that complicates its ultra-sensitive quantification.

Few methods for actinomycin D quantification relying on LC-MS have been previously reported; three for plasma [19,20,21] and one for dried blood spots [22]. Considering the utilized plasma amount, the lowest reported lower limit of quantification (LLOQ) is 0.5 ng/mL for actinomycin D quantification in plasma micro-samples of 30 µL originating from a pediatric population [19]. To accurately investigate the expected very low CNS disposition of actinomycin D in mice, we developed a rapid and simple, ultra-sensitive UPLC-MS/MS assay (LLOQ of 0.05 ng/mL) that requires only 20 µL of plasma, brain tissue homogenate, or microdialysate.

## 2. Materials and Methods

### 2.1. Pre-Clinical Mouse Studies

The experiments were carried out in accordance with the European regulations for care and use of laboratory animals (2010/63/EU) under the German license number G-187/18 and the internal reference number DKFZ374, approved by the responsible regional council (Regierungspräsidium Karlsruhe, Germany). Female 8-week-old NSG mice (NOD.Cg-Prkdc^scid^ Il2rg^tm1Wjl^/SzJ-NOD-SCID gamma-mice) with a weight of approximately 25 g were used for the studies. For microdialysis experiments, a microdialysis guide cannula was stereotactically implanted into the right striatum and stabilized with screws and dental acrylic cement. Mice received analgesia (metamizole) and anesthesia with isoflurane during surgery. After six days of recovery, the microdialysis procedure was initiated. A microdialysis probe (CMA7, 1 mm, molecular cut-off 55 kDa, CMA Microdialysis AB, Kist, Sweden) was inserted through the guide cannula and connected to a pump system operating at a constant flow rate of 0.5 µL/min Ringer-1 % bovine serum albumin (BSA) solution. Throughout the study, mice could freely move. Microdialysis samples were serially collected each hour. Because the number of blood collections in individual mice is limited, we estimated the optimal timepoints for a pharmacokinetic feasibility study by allometric simulation of actinomycin D’s pharmacokinetic profile in mice according to published data in humans [8,9]. For the allometric simulation, mouse plasma pharmacokinetic profiles were simulated by allometrically scaling the clearance and volumes parameters using classic exponents (0.75 for clearances and 1 for volumes) from mouse to human, based on 0.5 mg/kg dosage [23]. We aimed at assessing the log-linear profile of the terminal elimination phase, which appeared to start 0.5–0.75 h after injection in the allometric simulation (Figure S1). Therefore, we chose to take blood samples 0.75, 6, and 22 h after intravenous bolus injection into the tail vein for the assay’s feasibility pharmacokinetic experiment. Three animals received an intravenous bolus injection of 0.5 mg/kg actinomycin D in 0.125 mL per mouse (25 g) of a 0.1 mg/mL dosing solution. This dosage corresponds to the usual therapy protocols in humans according to allometric scaling [24]. Full blood samples were transferred into citrate tubes (Sarstedt, Germany) and centrifuged at 1600× *g* for 10 min to generate plasma samples, which were stored at −80 °C until analysis. For determination of actinomycin D disposition in the CNS, brain tissue, and plasma samples were harvested following euthanasia with increasing concentrations of CO_2_ at 2 h (three animals) or 22 h (four animals) after intravenous bolus injection of 0.5 mg/kg actinomycin D. Samples were stored at −80 °C until analysis. For validation of the UPLC-MS/MS quantification, blank plasma from 6 naïve mice was used and blank brain tissue was obtained from 2 unexposed mice. Whole brain tissue was homogenized using a Bead Ruptor 4 homogenizer (Omni International Inc, Kennesaw, GA, USA) in acetonitrile (ACN)/water (1/19, *v*/*v*) + 0.5 % Triton X and 0.1 % formic acid (FA) (100 mg brain tissue/mL) in 2.0 mL tubes containing ~25 glass beads (0.75–1 mm; Carl Roth GmbH, Karlsruhe, Germany) for 2 × 1 min.

### 2.2. Drugs, Chemicals, Solvents, and Materials

Actinomycin D (Lyovac Cosmegen^®^) for intravenous administration to mice was obtained from Recordati Rare Diseases Germany GmbH (Ulm, Germany), dissolved according to the manufacturer´s instructions using 1.1 mL water (B. Braun, Melsungen, Germany), and diluted with 0.9% normal saline solution (B. Braun) to a concentration of 0.1 mg/mL. Ringer solution was also obtained from B. Braun. Pure actinomycin D (98%; C_62_H_86_N_12_O_16_, 1255.4 g/mol) for analytical purposes and in vitro assays was purchased from Biozol Diagnostica Vertrieb GmbH (Eching, Germany) supplied by Toronto Research Chemicals (North York, ON, Canada). Actinomycin C (98%; C_63_H_88_N_12_O_16_, 1269.4 g/mol) was obtained from Santa Cruz Biotechnology (Dallas, TX, USA). Water, methanol (MeOH), ACN, and FA were purchased from Biosolve BV (ULC/MS grade; Valkenswaard, The Netherlands). Blank mouse plasma (CD-1) was obtained from Innovative Research (Novi, MI, USA). Heparin (Clexane, 4000 I.E., 40 mg/0.4ml) was purchased from EurimPharm Arzneimittel GmbH (Saaldorf-Surheim, Germany). Tween-20 was obtained from Merck KGaA (Darmstadt, Germany). TritonX100, polylysine, polyvinylpyrrolidone (PVP), culture media, medium supplements, and buffers were purchased from Sigma-Aldrich (Taufkirchen, Germany). Fetal calf serum (FCS) was purchased from Biochrom (Berlin, Germany) and crystal violet from AppliChem (Darmstadt, Germany). Vincristine was obtained from Biotrend (Cologne, Germany). Bovine serum albumin (fraction V; BSA) was purchased from Carl Roth (Karlsruhe, Germany).

### 2.3. Standard Solutions

The stock solution of the internal standard (IS) (actinomycin C) was prepared at 0.3 mg/mL in ACN/H_2_O (1/1, *v*/*v*) + 0.1% FA in a glass vial. The IS stock solution was diluted 200,000-fold with ACN + 0.1% FA to yield the solution utilized for protein precipitation at a concentration of 1.5 ng/mL. For preparation of calibration stock solutions, actinomycin D (1.10 mg) was weighed into 5 mL volumetric flasks, filled up with ACN/H_2_O (1/1, *v*/*v*) + 0.1% FA, and diluted to yield calibration sub-solutions at 0.2, 0.8, 2, 4, 12, 40, 120, and 400 ng/mL. Quality control (QC) sub-solutions were prepared accordingly from an independent weighing of actinomycin D (0.61 mg/5 mL) at 0.2, 0.6, 12, and 240 ng/mL.

### 2.4. Calibration and QC Samples

Plasma calibration samples were prepared by spiking blank plasma (75 µL) with 25 µL of the calibration sub-solution, yielding plasma concentrations of 0.05, 0.2, 0.5, 1, 3, 10, 30, and 100 ng/mL. Plasma QC samples were prepared accordingly at concentrations of 0.05, 0.15, 3, and 60 ng/mL. Brain tissue homogenate calibration samples were prepared by spiking brain tissue homogenate (75 µL), which was prepared at 100 mg tissue per mL of 5% aqueous ACN containing 0.5% Triton X, with 25 µL of the calibration sub-solution, yielding concentrations corresponding to 0.5, 2, 5, 10, 30, 100, 300, and 1000 ng/g. Brain tissue homogenate QC samples were prepared accordingly, at corresponding concentrations of 0.5, 1.5, 30, and 600 ng/g. Solutions were kept at 4 °C for up to 6 months.

### 2.5. Sample Preparation

For protein precipitation of plasma microsamples, an excess of ACN (50 µL) with 0.1% FA containing the IS was applied to an Impact^®^ 96-well protein precipitation plate (Phenomenex, Torrance, CA, USA). Subsequently, 20 µL of the plasma samples were added and the plates sealed and shaken for 1 min. Brain tissue homogenate samples were processed accordingly. For protein depletion, samples were filtered into a 96-well collection plate (Waters, Milford, MA, USA) by applying positive pressure with a 96-well positive pressure unit (Waters, Milford, MA, USA) operated with air overpressure of 5 psi. After addition of 40 µL of water containing 0.1% FA, the collection plates were sealed, shaken, and extracts injected onto the UPLC system.

### 2.6. Determination of Protein-Bound Fraction

To determine the previously unknown unbound fraction of actinomycin D in plasma and brain tissue homogenate, we performed rapid equilibrium dialysis (RED) in duplicate. For this, samples were spiked with drug solutions that were prepared in plasma or brain tissue homogenate to obtain minimally diluted samples (30 ng/mL for plasma and 30 ng/g for brain tissue). The free actinomycin D tissue fraction was also quantified in a pooled sample of brain tissue homogenates from the three mice euthanized 2 h after dosing. Determinations were performed on a RED apparatus (ThermoFisher Scientific, Waltham, MA, USA) according to the manufacturer´s instructions under gentle agitation for 12 h at 37 °C.

### 2.7. Instrumental Analysis Parameters

The quantification setup consisted of an Acquity UPLC^®^ System coupled to a Xevo TQ-XS triple-stage quadrupole mass spectrometer with an attached Z-spray heated electrospray ionization (ESI) source (Waters, Milford, MA, USA). A Waters Acquity UPLC^®^ Peptide BEH C18 column (300 Å, 1.7 μm, 2.1 × 50 mm) maintained at 60 °C was applied for chromatographic separation. The eluent consisted of 5% (v) ACN in water with 0.1% FA (eluent A) and ACN with 0.1% FA (eluent B). The flow at a rate of 0.5 mL/min was directly introduced into the ion source between 1.0 and 1.6 min after injection and otherwise directed to the waste. Gradient starting conditions of 60% A/40% B were kept for 0.5 min and the ratio subsequently changed to 100 % B within 1.5 min (0.5–2.0 min). These conditions were maintained for an additional 0.5 min. The ratio was then returned to starting conditions over a period of 0.5 min. While the Sample Manager prepared the following injection, which lasted about 1 min, the starting conditions were kept. Samples were maintained at 15 °C while in the Sample Manger. After injection, the needle was flushed with ACN/water/MeOH (2/1/1, *v*/*v*/*v*) + 1% FA. As a result, a complete injection cycle took 4 min. The injected sample volume was 20 µL. ESI source parameters were manually optimized to a capillary voltage of 2500 V, source temperature of 150 °C, cone gas flow (N_2_) of 50 L/h, desolvation gas flow (N_2_) of 1000 L/h, and desolvation temperature of 600 °C. The Xevo TQ-XS was tuned to actinomycin D and actinomycin C for selective reaction monitoring (SRM) measurements, which were performed with CID in positive ion mode using 0.15 mL/min of argon, using the IntelliStart procedures integrated into the MassLynx V4.2 system software. This resulted in an optimized cone voltage of 100 V and a collision energy of 62 V. Monitored mass transitions were *m/z* 1255.6 → 459.1 for actinomycin D and *m/z* 1269.6 → 459.1 for actinomycin C.

### 2.8. Validation of the Analytical Methods

The assay was validated according to the FDA´s and EMA´s published recommendations [25,26], independently for all analyzed biological matrices. Full validation was performed for plasma and brain tissue homogenate. For the transfer of the assay to microdialysate partial validation with a single validation run was considered sufficient, because the Ringer´s buffer containing BSA constitutes a simplified plasma matrix. In addition, actinomycin D cell homogenate quantification was validated accordingly with also a single validation run, because we regarded the cell homogenate as a simplified tissue homogenate matrix.

Accuracy was calculated as the percentage of the ratio of the mean determined concentration in individual analytical runs and the nominal value. Precision was expressed in percent and is defined as the coefficient of variation of the measured sample concentrations at each QC level. Three validation runs were analyzed, each containing eight calibration samples in duplicate determination and four QC samples at LLOQ, and low, mid, and high QC concentration in six replicates. Selectivity was evaluated using blank plasma samples from six naïve mice and brain tissue homogenates from two mice. Extraction recovery was calculated from peak areas after extraction from the QC samples divided by the respective peak areas obtained from blank plasma spiked after extraction. Matrix effects were evaluated by comparison of the peak areas of the blank plasma samples spiked after extraction with the respective peak areas of matrix-free UPLC solvent samples spiked with the identical amount [27]. Stability of the analyte was tested in three freeze-and-thaw cycles separated by at least 24 h, in plasma samples stored for 1 month at −25 °C, in microdialysate stored at room temperature for 2 d, and in extracts left in the autosampler for 24 h. Additionally, an incurred sample reanalysis was performed for all plasma samples with sufficient volume for reanalysis.

### 2.9. Verification of Actinomycin D as a Substrate of P-gp

To confirm the P-gp substrate characteristics of actinomycin D, we conducted growth inhibition and uptake assays in a cell line over-expressing human P-gp/*ABCB1* (L-MDR1 cells) in comparison to the respective parental cell line (LLC-PK1). L-MDR1 cells were kindly provided by A. H. Schinkel (The Netherlands Cancer Institute, Division of Experimental Therapy, Amsterdam, The Netherlands). The cells were cultured under standard cell culture conditions with medium M199 supplemented with 10% FCS, 2 mM glutamine, 100 U/mL penicillin, and 100 µg/mL streptomycin sulfate. The culture medium for L-MDR1 was supplemented with 0.64 µM vincristine to maintain P-gp/*ABCB1* expression. One day before using the cells in the assays, both cell lines were fed with vincristine-free culture medium.

Growth inhibition assays in L-MDR1 and LLC-PK1 cells were conducted to evaluate whether actinomycin D is less effective in P-gp over-expressing cells, indicating reduced influx in the presence of P-gp. The assays were performed in quadruplicate, as described previously [28], using ten concentrations of actinomycin D from 0.0005–10 µM in octuplets.

For the uptake assays, cells were incubated in the dark at 37 °C on a rotary shaker in cell culture medium with 0.01 µM actinomycin D for 10, 30, 60, or 120 min in duplets, each containing 1 million cells. After the incubation period, cells were washed twice with ice-cold PBS. The dry pellet was lysed using 100 µL of 0.1 M aqueous HCl and homogenized using sonification for 5 min. Cell homogenates were processed according to plasma sample preparation.

### 2.10. Calculations and Statistical Methods

Calibration curves were determined with 1/x^2^ weighted linear regression using peak area ratios of the analyte to IS. This calculation was performed using the software TargetLynx V4.2 (Waters, Milford, MA, USA). Plasma pharmacokinetics were determined with the software Kinetica (v 5.0; Thermo Fisher Scientific, Waltham, MA, USA). Standard calculations were performed using Microsoft Office Excel 2010 (Mountain View, CA, USA). Concentration–response curves and IC_50_ values were calculated by GraphPad Prism version 9.0.0 (GraphPad Software Inc., La Jolla, CA, USA) according to a sigmoid E_max_ model.

## 3. Results and Discussion

### 3.1. Performance of the UPLC-MS/MS Assay

#### 3.1.1. Mass Spectrometric and Chromatographic Characteristics

In the positive ESI of actinomycin D, two main signals were observed, the [M+2H]^2+^ ion at *m/z* 628.6 and the [M+H]^+^ signal at *m/z* 1255.6. The doubly charged species showed the highest intensity at medium cone voltage and substantially exceeded the maximum intensity of the singly charged species, which showed its highest intensity at a high cone voltage. In the CID of the [M+H]^+^ precursor, the most abundant fragment was found at *m/z* 459.1, followed by a signal at *m/z* 300.2, while for the [M+2H]^2+^ signal, the most prominent fragments occurred at *m/z* 300.2 and *m/z* 203.1. When determined in solution, the fragments of the doubly charged species exhibited a significantly higher intensity than the CID fragments of the singly charged signal. However, when evaluating the mass transitions in plasma extracts, the transition of *m/z* 1,255.6 459.1 produced the most intense signal, which was therefore chosen for SRM. Actinomycin D undergoes a complex fragmentation in CID consisting of multiple consecutive reactions and rearrangements, which were previously noted by Thomas and co-workers [29]. The structure of the monitored fragment at *m/z* 459.1 is shown in Figure 1.

**Figure 2:**
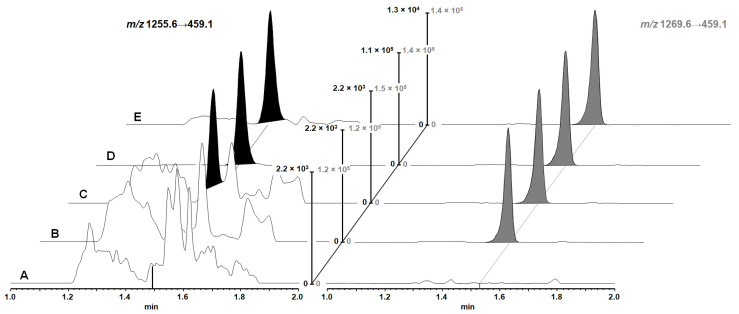
Selected UPLC-MS/MS chromatograms of the processed brain tissue homogenate samples, with the analyte transition in black and internal standard (IS) transition in grey: (A) blank sample, (B) sample with added IS, (C) sample at a lower limit of quantification (LLOQ) level (representing 0.050 ng/mL), (D) sample at a mid QC concentration (representing 3.00 ng/mL), and (E) brain tissue sample 2 h after intravenous administration of 0.5 mg/kg actinomycin D to mouse #1 (calculated actinomycin D concentration 0.409 ng/mL corresponding to 4.09 ng/g due to tissue homogenization at 0.1 g/mL). The intensity of the blanks was normalized to the value of the analyte peak in the LLOQ chromatogram while the intensity in the remaining chromatograms was normalized to the analyte peak with the IS and analyte transition processed independently.

For chromatography optimization we tested reverse phase columns (C18 and C4) due to actinomycin D´s high lipophilicity. We used reverse phase material with a large pore width of 300 Å because large pore widths usually foster optimal mass transfer kinetics for macromolecules. Chromatographic peak shape and separation performed better on the C18 column. In accordance with the high lipophilicity of actinomycin D, a gradient from 40–100% eluent B was found optimal and yielded peaks with a width at baseline of 6 s (Figure 2).

#### 3.1.2. Choice of Internal Standard

Because an isotopically labelled analogue of actinomycin D was not available, and in accordance with our own tests (data not shown) and the conclusion of Damen and co-workers [19] that 7-amino actinomycin D is not suitable for use as IS due to the large hydrophilic shift in retention time (and maybe due to the inequality of ionization and fragmentation characteristics), we aimed at the use of a structural analogue of actinomycin D with similar chromatographic characteristics.

Actinomycin C differs from actinomycin D by the exchange of a valine to isoleucine in one of the two comprised cyclodepsipeptides, which results in an additional methyl group. Hence, it possesses very similar physicochemical properties to actinomycin D with only a minimal lipophilic shift, which was not baseline-separated under the conditions used in our UPLC separation (Figure 2). Therefore, actinomycin C was expected to best reflect the matrix effects of actinomycin D that occur in the sample analyses, in part because the identical fragment is monitored in SRM (*m/z* 1269.6 → 459.1); for all these reasons, actinomycin C was chosen as the IS.

#### 3.1.3. Sample Preparation and Extraction Characteristics

Quantification of actinomycin D was performed in micro-samples (20 µL) of plasma, brain tissue homogenate, and cerebral microdialysate. Micro-sampling is a prerequisite for quantification of cerebral microdialysis samples and pharmacokinetic studies in small animals. It is also beneficial for pediatric blood sampling. In our experiments, we used whole brain homogenate determinations. However, in principle, quantification in brain homogenate microsamples facilitates assessment of actinomycin D brain distribution on a small scale, e.g., in limited tumor material from patient-derived mouse xenografts or patient biopsies. In the analysis of brain tissue samples, we standardized the proportion of tissue in the homogenates (0.1 g/mL) to avoid inconsistencies regarding the matrix effects between samples. As a consequence, the measured concentrations in homogenates (ng/mL) correspond to 10-fold higher brain tissue concentrations expressed in ng/g.

Efficient extraction of actinomycin D was achieved by protein precipitation with a 2.5-fold excess of ACN + 0.1% FA (50 µL). For the monitored mass transitions of the analyte and IS, no interference was observed, facilitating sensitive quantification. Recoveries were consistent across the QC and IS concentrations and were ≥91% for the plasma and brain tissue homogenate (Table S1).

#### 3.1.4. Validation Results

The extraction by protein precipitation combined with UPLC-MS/MS quantification for actinomycin D fully met the FDA and EMA requirements for bioanalytical method validation [25,26] for all investigated biological matrices. Selectivity of the assay was demonstrated by the absence of interfering signals in the plasma from six individual mice and brain tissue homogenates from two individual mice not exposed to actinomycin D (Figure 2). Within the calibrated range (0.05–100 ng/mL), correlation coefficients (r^2^) of ≥0.99 were obtained using linear regression and a 1/x^2^ weighting (Table S2). Tables S3 to S6 give an overview of the intraday and interday accuracies and precision values of the assays, which were well within the required limits. Stability of actinomycin D during freeze-and-thaw cycles and in plasma samples at −25 °C for 1 month was confirmed by accuracies for a low and high QC of 87.6–93.6% and 95.6–100.7%, respectively. Long-term stability in human plasma (7 months at −20 °C) has already been demonstrated [19]. Extracts were stable over the course of individual analyses, as evidenced by accurate reanalysis of low and high QC samples that remained in the autosampler for 24 h. Actinomycin D in solution was stable for at least 6 months, as shown by comparison of the quantification results of freshly prepared QC samples with stored calibration solutions, which yielded accuracies ranging from 86.4–88.3%. Table S7 gives an overview of the obtained stability data during validation. Analytical variability observed in the incurred sample reanalysis was within the allowed limits, and all reanalyzed samples showed a deviation of ≤16.2% (Table S8).

In addition, we demonstrated the feasibility of our spiking process by the accurate determination of minimally diluted QC plasma samples (with spike solutions prepared in the plasma already) with calibration samples prepared with spike solutions, which resulted in accuracies of 89.8 to 94.7% (Table S9).

#### 3.1.5. Matrix Effect

Endogenous substances, especially peptides and phospholipids, are expected to interfere in the SRM analysis of precipitated plasma samples of actinomycin D. However, due to the efficient chromatographic separation, no interfering signals were observed in six individual mouse blank plasma samples, demonstrating the selectivity of the assay. Furthermore, there were no interfering signals in the brain tissue homogenate of two unexposed mice and in their pooled homogenate. Matrix effects of low to high QC samples were consistent, reproducible, and ranged between −33.8 and −25.8% (corresponding to IS-normalized values of 102.0 to 109.8%) for plasma, and between −44.0 and −36.8% (corresponding to IS-normalized values of 110.3 to 114.5%) for brain tissue homogenate (Table S10). Because previous reports have not assessed the IS-normalized matrix effects and absolute matrix effects have only been investigated qualitatively with the different post-infusion technique, an accurate comparison with previously established methods was considered infeasible. However, the compliance of the IS-normalized matrix effects of our assay with the required limits for bioanalytical method validation (100 ± 15%) demonstrates the suitability of actinomycin C for use as IS due to sufficient balancing of the occurring matrix effects.

### 3.2. Plasma Concentrations of Actinomycin D in Mice after Intravenous Injection

**Figure 3:**
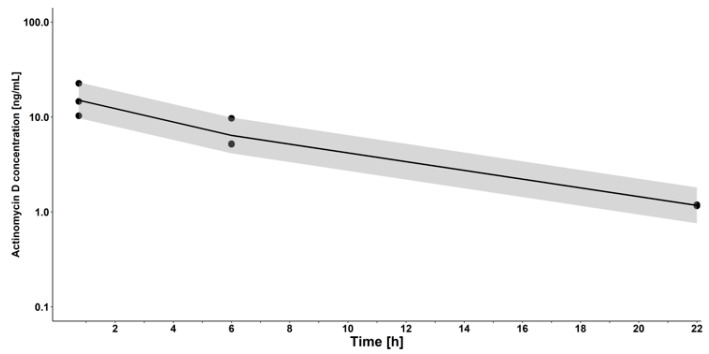
Plasma concentration–time profiles of intravenous actinomycin D (0.5 mg/kg) in three mice.

Initially, the applicability of the assay was demonstrated by determination of individual plasma concentrations of three mice receiving intravenous actinomycin D. Due to the low sample volume required for quantification, the plasma concentration–time profiles could be assessed individually (Figure 3), reducing the number of animals required for reliable pharmacokinetic evaluation.

The pharmacokinetic parameters are shown in Table 1. However, these must be regarded as estimates, due to the limited samplings available, especially due to actinomycin D´s complex pharmacokinetics matching a three-compartment model in humans [9,11]. The calculated AUC agreed well with previously determined values in mice [13]. However, the determined half-life in our study was 3-fold longer. This difference is probably the result of the more sensitive determination of the actinomycin D plasma concentration over a considerably longer period in our experiments (22 h vs. 6 h), reducing the potential over-estimation of the intermediate half-life.

Because there is no reliable information on the protein binding of actinomycin D, we additionally determined the unbound fraction of the drug in mouse plasma, which was 23.1%.

### 3.3. Setup of the Microdialysis Experiments

**Figure 4:**
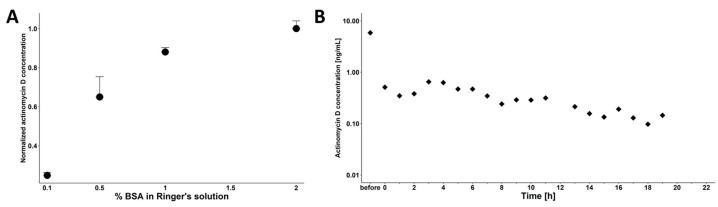
(**A**) Actinomycin D showed nonspecific binding to the microdialysate equipment, which can be reduced by adding bovine serum albumin to the Ringer solution. (**B**) Actinomycin D concentration detected in in vivo cerebral microdialysis samples of a non-dosed mouse after prior in vitro calibration of the microdialysis probe with actinomycin D.

In preparation of the cerebral microdialysis, we first investigated the adsorption and recovery characteristics of the microdialysis probes, tubing, and pump system, to test the validity of the microdialysis determinations. In vitro, non-specific binding and analyte recovery studies revealed that actinomycin D substantially adhered to the polypropylene equipment parts, such as the microdialysis sampling tubes. Additionally, actinomycin D bound nonspecifically to the glass of the syringes integrated in the microdialysis pump system. Coating strategies using bovine serum albumin (BSA), polylysine, Triton x, Tween-20, heparin, and PVP of the experimental system (polypropylene) and glass surfaces were unsuccessful (Figure S2). In order to reduce nonspecific binding, BSA supplement of the microdialysis buffer was tested in different concentrations, ranging from 0.1 to 2%. BSA additions ≥1% were sufficient to quantitatively reduce nonspecific binding (Figure 4A). Therefore, 1% BSA in Ringer solution was used for all microdialysis experiments. A 1-mm-long polyestersulfone probe (showing the best recovery of actinomycin D; Table S11) with a cutoff of 55 kDa was chosen for microdialysis studies operated at a low flow rate of 0.5 µL/min. For each microdialysis probe, in vitro recovery studies were performed using an actinomycin D stock solution of 100 ng/mL prepared in 1% BSA Ringer solution in order to enable the back-calculation of the microdialysis concentrations to brain concentrations. Probe performance tests showed a median in vitro retrodialysis recovery coefficient, corresponding to the ratio of drug penetrating the probe, of 84.5 ± 5.4% (Table S12). Additionally, actinomycin D was found stable for 2 d in the used buffer (Table S7), which is a prerequisite for microdialysis experiments.

### 3.4. Cerebral Microdialysis Measurements in Healthy Mice

Cerebral microdialysis allows for individual assessment of CNS drug disposition over time, thereby reducing the number of required animals within pharmacokinetic studies. The established microdialysis setup was first tested in an unexposed mouse to ensure unambiguous detection of administered actinomycin D. However, despite BSA addition to the used buffer, cleaning of the syringes with ethanol and H_2_O, overnight rinsing of the probe with Ringer solution, and replacement of the entire tubing system, the drug was detected in an unexposed mouse (Figure 4B), indicating nonspecific binding to the microdialysis system, possibly also involving the probe membrane.

To avoid contamination of the probe during calibration with the drug of interest, the IS Actinomycin C with its similar physicochemical properties was used for the necessary recovery studies. This strategy ensures that the microdialysis setup is accurately calibrated for recovery calculations without pre-exposing the system to the analyte of interest, thus ensuring unambiguous discrimination from the drug administered to the animal. Concentration-dependent in vitro recovery studies using actinomycin C showed an average in vitro retrodialysis coefficient of 87.6 ± 0.5% well matching the results obtained with actinomycin D (Table S13).

To support the calibration procedure with actinomycin C, the quantification assay was adapted to allow measurements of actinomycin C in the microdialysate in a concentration range from 0.1 to 100 ng/mL. To this end, we have exchanged the role of analyte (→ actinomycin C) and IS (→ actinomycin D). Because the assay was very similar to the quantification of actinomycin D, the assay was validated for the quantification of actinomycin C using a single validation batch, fulfilling the pertinent requirements, the results of which are shown in Table S14.

**Figure 5:**
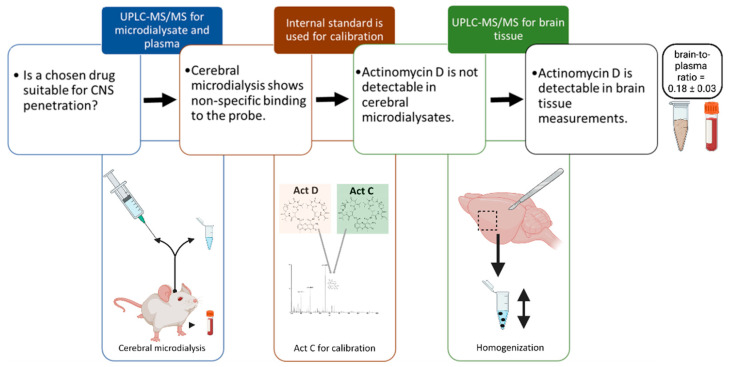
Schematic workflow of the methodology for determining the central nervous system (CNS) drug disposition of actinomycin D (Act D). Despite the use of actinomycin C (Act C) for probe calibration and avoiding nonspecific binding of actinomycin D to the microdialysis equipment, only whole brain tissue measurements were suitable for measurement of the drug´s brain disposition. Created with BioRender (Biorender AG, Münchwilen, Switzerland).

This strategy was subsequently validated in vivo. When the calibration was performed with actinomycin C, no actinomycin D was detectable in the cerebral microdialysis samples of an unexposed mouse, demonstrating the applicability of the strategy. Because the IS for actinomycin D quantification (actinomycin C) is used in a sufficiently high concentration, the remaining actinomycin C in the microdialysis system does not influence actinomycin D quantification. However, no detectable concentrations of actinomycin D were measured in the CNS microdialysate of a dosed mouse while quantification of the plasma samples revealed the expected drug concentration–time profile (data not shown). Evaluations based on cerebral microdialysis, therefore, do not appear to be feasible for the assessment of CNS disposition of actinomycin D until even more sensitive analytical methods become available, making measurements of tissue homogenate the currently available option for detection of the drug at the site of interest. The scheme of the followed workflow is shown in Figure 5.

Nevertheless, the established strategy of incorporating BSA into the microdialysis buffer and using the IS of the quantification assay to calibrate the microdialysis probes (ideally: isotope-labelled compounds) represents a viable general approach for cerebral microdialysis measurements of therapeutics and particularly for highly lipophilic drugs that should be further tested.

### 3.5. Determination of Actinomycin D CNS Concentrations after Intravenous Bolus Injection to Mice

Brain tissue measurements were performed after cleaning the organ via cardiac perfusion with 20 mL of PBS to prevent contamination from the drug retained in brain capillaries. Figure 3 depicts the obtained plasma and brain tissue profiles.

Two hours after intravenous actinomycin D injection, the mean plasma concentration was 22.7 ng/mL and the corresponding brain tissue concentration reached 4.1 ng/g (Table 2). Due to the thorough cardiac perfusion with PBS and the fact that the mouse brain contains only 6% blood [30], the brain tissue concentration found cannot be explained by blood contamination (the measured value substantially exceeds the maximum value reachable via blood contamination, which is 1.3 ng/g) and therefore appears to reliably reflect actinomycin D brain concentration. Therefore, these findings, with the concurrently undetectable unbound fraction of actinomycin D in the microdialysis experiments, suggest that the unbound fraction of actinomycin D in brain tissue (and most likely also in other tissues) is very low and substantially smaller than the corresponding plasma concentrations, which is consistent with its tight binding to DNA. The investigation of the free fraction of these brain tissue homogenates revealed a low free fraction that could not be exactly determined because the peak areas in the samples of the buffer of the rapid equilibrium device (corresponding to the unbound concentration) were well below the LLOQ peak areas. However, if the response values were evaluated, the free fraction was estimated to be <3%. These results matched those of minimally diluted spiked brain tissue homogenate, which showed a free fraction of 3.4%.

The obtained brain-to-plasma ratio of 0.18 is somewhat higher than suggested in a previous report [10]. However, in this earlier study, actinomycin D was quantified in cerebrospinal fluid three hours after intravenous administration, i.e., in a protein-poor compartment that is also separated from blood and tissue by active barriers. In both reported patients, the cerebrospinal fluid concentrations reached approximately 10% of the corresponding plasma concentrations, which is also substantial assuming that the unbound fraction in humans is similarly low as in mice. In a more recent study in mice [13], the disposition of actinomycin D at a dose of 0.5 mg/kg (similar to our study) was investigated by tissue measurements. However, the measured brain concentrations were only evaluated as peak areas of the utilized quantification method due to sample volume restrictions, and hence, accurate brain concentrations could not be established.

Our experiments revealed an increase in the brain-to-plasma ratio over time, and 22 h after intravenous administration of actinomycin D it reached a mean value of 0.53 (Table 2). The observed slower elimination from brain tissue is concordant with the lower free drug concentration detected in brain tissue homogenate compared to plasma.

**Figure 6:**
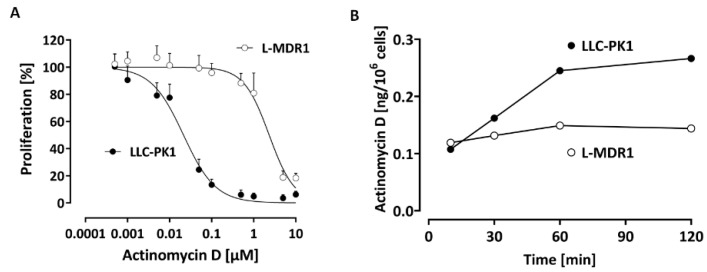
(**A**) Concentration-dependent effect of actinomycin D on the proliferation of the P-gp over-expressing cell line L-MDR1 and the corresponding parental cell line LLC-PK1. Each curve depicts the results of four experiments with each concentration tested in octuplet. Data are expressed as the mean ± SD for n = 32 wells. The IC_50_ in L-MDR1 cells was 2.4 ± 0.6 µM and in LLC-PK1 cells 0.021 ± 0.006 µM (>100-fold difference). (**B**) Time-dependent uptake of 0.01 µM actinomycin D in the P-gp over-expressing cell line L-MDR1 in comparison to the parental cell line LLC-PK1. Each data point depicts the mean of a duplicate determination.

Previous [13] and our in vitro experiments clearly demonstrate much greater resistance and lower accumulation of actinomycin D in cell lines over-expressing P-gp (Figure 6). This suggests that actinomycin D is indeed a P-gp substrate.

Nevertheless, it was found that the detected brain exposure of actinomycin D in P-gp knockout mice exceeded that of wildtype mice by a modest 2.3-fold [13]. Studies with other prototypical P-gp substrates demonstrated that brain concentrations in P-gp-knockout mice increase much more compared with wildtype mice, e.g., 35-fold for digoxin and 87-fold for ivermectin [31]. Moreover, clinical data indicate no relevant influence of functional *ABCB1* polymorphisms on the exposure with actinomycin D in children [8], questioning the profound modulation of actinomycin D pharmacokinetics by P-gp in vivo. This is also consistent with our data in mice showing that actinomycin D is not completely prevented from crossing the BBB by P-gp, which may be a consequence of the low free fraction in the tissue.

Our investigation is the first to accurately determine actinomycin D brain tissue concentrations, revealing considerable penetration of the drug across the BBB. The determined brain tissue concentration after 2 and 22 h correspond to approximately 3 nM and 1.2 nM, which exceeds the EC_50_ value of actinomycin D-sensitive brain tumor cell lines [15,18] approximately by 6-fold and 2-fold. The measured total tissue concentration may be effective due to actinomycin D´s mechanism of action relying on DNA binding.

## 4. Conclusions

We have successfully established an experimental framework to study the CNS disposition of actinomycin D that concurrently suggests general solutions to key challenges in measuring cerebral microdialysis samples. The evaluation of CNS concentration was supported by a newly developed ultra-sensitive UPLC-MS/MS assay for actinomycin D quantification in micro-samples, with an LLOQ of 0.05 ng/mL, which is one order of magnitude more sensitive than the previous methods while requiring smaller sample volumes. Actinomycin D was adsorbed nonspecifically and to a relevant extent to the microdialysis equipment, resulting in apparent drug exposure of the non-dosed mice. This underscores the importance of confirming the validity of the experimental microdialysis setup before applying it in vivo, because most drugs potentially available to the CNS are highly lipophilic and thus tend to be highly adsorbed, especially in aqueous buffers. The adsorption challenges were solved by using a BSA-Ringer solution in the microdialysis system and by using the IS to calibrate the required individual recovery efficiencies of the microdialysis probes. This strategy represents a generally applicable methodology for (cerebral) microdialysis experiments of highly hydrophobic therapeutics. However, in mice receiving an allometrically scaled therapeutic dose, actinomycin D could not be detected in the microdialysate, whereas brain tissue concentrations were readily detectable. Consequently, actinomycin D is not suitable for cerebral microdialysis experiments because the tissue does not release it adequately, probably due to high tissue binding, most likely to its abundant target nuclear DNA. Therefore, brain tissue homogenate measurements are favored when evaluating concentrations of actinomycin D in the CNS. Investigations in mice revealed substantial penetration of the BBB by intravenous actinomycin D, reaching a potentially effective tissue concentration for an extended period of at least 22 h. These results indicate that actinomycin D might be a suitable candidate for further development as a treatment for pediatric CNS neoplasia. However, methods of either reducing peripheral (systemic) actinomycin D toxicity or increasing BBB penetration are likely required for effective treatment.

## Figures and Tables

**Table 1:** Single-dose pharmacokinetics of intravenous actinomycin D in mice.

Mouse	C_max_ (ng/mL)	AUC (ng/mL × h)	V_ss_ (L)	Cl (mL/min)	t_1/2_ (h)
1	22.6	171	0.53	1.22	5.07
2	14.6	111	0.98	1.87	6.14
3	10.3	101	1.23	2.06	6.90
Mean	15.8 ± 5.1	128 ± 31	1.10 ± 0.35	2.06 ± 0.43	6.04 ± 0.75

AUC: area under the concentration–time curve extrapolated to infinity; Cl: clearance; C_max_: maximal plasma concentration; t_1/2_: half-life; V_ss_: volume of distribution.

**Table 2:** Plasma and brain concentrations of mice 2 and 22 h after intravenous administration of 0.5 mg/kg actinomycin D.

Time (h) after Administration	Mouse	Plasma Actinomycin D (ng/mL)	Brain Actinomycin D (ng/g)	Brain-to-Plasma Ratio
2	1	19.2	4.09	0.21
	2	26.8	4.77	0.18
	3	22.0	3.55	0.16
	Mean	22.7 ± 3.9	4.14 ± 0.50	0.18 ± 0.03
22	4	1.95	1.56	0.80
	5	5.79	2.15	0.37
	6	2.61	0.46	0.18
	7	3.24	2.49	0.77
	Mean	3.40 ± 1.45	1.67 ± 0.77	0.53 ± 0.26

## Data Availability

Data is available from the corresponding author upon reasonable request.

## Supplementary Materials

The following supplementary data on validation, stability investigations, and in vitro dialysis is available online at https://www.mdpi.com/article/10.3390/pharmaceutics13091498/s1, Figure S1: Allometric simulation of actinomycin D´s pharmacokinetic profile in mice, Figure S2: Coating strategies to avoid non-specific binding of actinomycin D, Table S1: Recovery data, Table S2: Calibration curves of validation batches, Table S3: Summary of quality control results for plasma actinomycin D, Table S4: Summary of quality control results for actinomycin D in brain tissue homogenate, Table S5: Summary of quality control results for actinomycin D in micro-dialysate, Table S6: Summary of quality control results for actinomycin D in cell homogenate, Table S7: Stability data of the plasma validation, Table S8: Results of the incurred sample reanalysis, Table S9: Summary of quality control results for actinomycin D quantification of minimally diluted QC samples, Table S10: Matrix effect data, Table S11: In vitro probe performance comparison, Table S12: Actinomycin D in vitro retrodialysis results, Table S13: Actinomycin C in vitro retrodialysis results, Table S14: Summary of quality control results for actinomycin C in micro-dialysate.

## References

[1] US National Cancer Institute, Dactinomycin (2019). https://www.cancer.gov/about-cancer/treatment/drugs/dactinomycin.

[2] Falini B., Brunetti L., Martelli M.P. (2015). Dactinomycin in NPM1-Mutated Acute Myeloid Leukemia. N. Engl. J. Med..

[3] Reich E., Franklin R.M., Shatkin A.J., Tatum E.L. (1961). Effect of Actinomycin D on Cellular Nucleic Acid Synthesis and Virus Production. Science.

[4] Reich E., Franklin R., Shatkin A., Tatum E. (1962). Action of Actinomycin D on Animal Cells and Viruse. Proc. Natl. Acad. Sci. USA.

[5] Sobell H.M. (1985). Actinomycin and DNA transcription. Proc. Natl. Acad. Sci. USA.

[6] Trask D.K., Muller M.T. (1988). Stabilization of type I topoisomerase-DNA covalent complexes by actinomycin D. Proc. Natl. Acad. Sci. USA.

[7] Wassermann K., Markovits J., Jaxel C., Capranico G., Kohn K.W., Pommier Y. (1990). Effects of morpholinyl doxorubicins, doxorubicin, and actinomycin D on mammalian DNA topoisomerases I and II. Mol. Pharmacol..

[8] Hill C.R., Cole M., Errington J., Malik G., Boddy A.V., Veal G.J. (2014). Characterisation of the clinical pharmacokinetics of actinomycin D and the influence of ABCB1 pharmacogenetic variation on actinomycin D disposition in children with cancer. Clin. Pharmacokinet..

[9] Mondick J.T., Gibiansky L., Gastonguay M.R., Skolnik J.M., Cole M., Veal G.J., Boddy A.V., Adamson P.C., Barrett J.S. (2008). Population Pharmacokinetic Investigation of Actinomycin-D in Children and Young Adults. J. Clin. Pharmacol..

[10] Tattersall M., Sodergren J., Sengupta S., Trites D., Modest E., Frei E. (1975). Pharmacokinetics of actinomycin D in patients with ma-lignant melanoma. Clin. Pharmacol. Ther..

[11] Veal G.J., Cole M., Errington J., Parry A., Hale J., Pearson A.D. (2005). Pharmacokinetics of dactinomycin in a pediatric patient population: A United Kingdom Children’s Cancer Study Group Study. Clin. Cancer Res..

[12] Walsh C., Bonner J.J., Johnson T.N., Neuhoff S., Ghazaly E.A., Gribben J.G. (2016). Development of a physiologically based phar-macokinetic model of actinomycin D in children with cancer. Br. J. Clin. Pharmacol..

[13] Hill C.R., Jamieson D., Thomas H.D., Brown C.D., Boddy A.V., Veal G.J. (2013). Characterisation of the roles of ABCB1, ABCC1, ABCC2 and ABCG2 in the transport and pharmacokinetics of actinomycin D in vitro and in vivo. Biochem. Pharmacol..

[14] Schinkel A.H. (1999). P-Glycoprotein, a gatekeeper in the blood–brain barrier. Adv. Drug Deliv. Rev..

[15] Tzaridis T., Milde T., Pajtler K.W., Bender S., Jones D.T.W., Muller S., Wittmann A., Schlotter M., Kulozik A.E., Lichter P. (2016). Low-dose Actinomycin-D treatment re-establishes the tumoursuppressive function of P53 in RELA-positive ependymoma. Oncotarget.

[16] Taylor J.T., Ellison S., Pandele A., Wood S., Nathan E., Forte G., Parker H., Zindy E., Elvin M., Dickson A. (2020). Actinomycin D downregulates Sox2 and improves survival in preclinical models of recurrent glioblastoma. Neuro-Oncol..

[17] Rusert J.M., Juarez E.F., Brabetz S., Jensen J., Garancher A., Chau L.Q., Tacheva-Grigorova S.K., Wahab S., Udaka Y.T., Finlay D. (2020). Functional Precision Medicine Identifies New Therapeutic Candidates for Medulloblastoma. Cancer Res..

[18] Schmidt C.A., Schubert N., Brabetz S., Mack N., Schwalm B.A., Chan J., Selt F., Herold-Mende C., Witt O., Milde T. (2017). Preclinical drug screen reveals topotecan, actinomycin D, and volasertib as potential new therapeutic candidates for ETMR brain tumor patients. Neuro-Oncol..

[19] Damen C.W.N., Israels T., Caron H.N., Schellens J.H., Rosing H., Beijnen J.H. (2009). Validated assay for the simultaneous quantification of total vincristine and actinomycin-D concentrations in human EDTA plasma and of vincristine concentrations in human plasma ultrafiltrate by high-performance liquid chromatography coupled with tandem mass spectrometry. Rapid Commun. Mass Spectrom..

[20] Veal G.J., Errington J., Sludden J., Griffin M.J., Price L., Parry A., Hale J., Pearson A.D., Boddy A.V. (2003). Determination of anti-cancer drug actinomycin D in human plasma by liquid chromatography–mass spectrometry. J. Chromatogr. B.

[21] Lee J.I., Skolnik J.M., Barrett J.S., Adamson P.C. (2007). A sensitive and selective liquid chromatography-tandem mass spectrometry method for the simultaneous quantification of actinomycin-D and vincristine in children with cancer. J. Mass Spectrom..

[22] Damen C.W.N., Rosing H., Schellens J.H.M., Beijnen J.H. (2009). Application of dried blood spots combined with high-performance liquid chromatography coupled with electrospray ionisation tandem mass spectrometry for simultaneous quantification of vincristine and actinomycin-D. Anal. Bioanal. Chem..

[23] Boxenbaum H. (1984). Interspecies Pharmacokinetic Scaling and the Evolutionary-Comparative Paradigm. Drug Metab. Rev..

[24] US Department of Health and Human Services, Food and Drug Administration, Center for Drug Evaluation and Research (CDER) (2005). Guidance for Industry, Estimating the Maximum Safe Starting Dose in Initial Clinical Trials for Therapeutics in Adult Healthy Volunteers. https://www.fda.gov/regulatory-information/search-fda-guidance-documents/estimating-maximum-safe-starting-dose-initial-clinical-trials-therapeutics-adult-healthy-volunteers.

[25] (2011). Committee for Medicinal Products for Human Use, European Medicines Agency, Guideline on Bioanalytical Method Valida-tion. https://www.ema.europa.eu/en/documents/scientific-guideline/guideline-bioanalytical-method-validation_en.pdf.

[26] US Food and Drug Administration (FDA) (2018). Guidance for Industry: Bioanalytical Method Validation. https://www.fda.gov/files/drugs/published/Bioanalytical-Method-Validation-Guidance-for-Industry.pdf.

[27] Matuszewski B.K., Constanzer M.L., Chavez-Eng C.M. (2003). Strategies for the Assessment of Matrix Effect in Quantitative Bioanalytical Methods Based on HPLC−MS/MS. Anal. Chem..

[28] Peters T., Lindenmaier H., Haefeli W.E., Weiss J. (2005). Interaction of the mitotic kinesin Eg5 inhibitor monastrol with P-glycoprotein. Naunyn-Schmiedeberg’s Arch. Pharmacol..

[29] Thomas D., Morris M., Curtis J.M., Boyd R.K. (1995). Fragmentation mechanisms of protonated actinomycins and their use in structural determination of unknown analogues. J. Mass Spectrom..

[30] Chugh B.P., Lerch J.P., Yu L.X., Pienkowski M., Harrison R.V., Henkelman R.M., Sled J.G. (2009). Measurement of cerebral blood volume in mouse brain regions using micro-computed tomography. NeuroImage.

[31] Schinkel A.H., Wagenaar E., Van Deemter L.A., Mol C., Borst P. (1995). Absence of the mdr1a P-Glycoprotein in mice affects tissue distribution and pharmacokinetics of dexamethasone, digoxin, and cyclosporin A. J. Clin. Investig..

