# Supplementary material for: Investigating the Central Nervous System Disposition of Actinomycin D: Implementation and Evaluation of Cerebral Microdialysis and Brain Tissue Measurements Supported by UPLC-MS/MS Quantification
Source: Pharmaceutics. 2021 Sep 17;13(9):1498. doi: 10.3390/pharmaceutics13091498 (PMC8466300; doi:10.3390/pharmaceutics13091498)
Supplement: Supplementary file 1 [file pharmaceutics-13-01498-s001.zip › pharmaceutics-1366606-supplementary.pdf]

pharmaceutics

MDPI

Supplementary Materials: Investigating the Central Nervous System Disposition of Actinomycin D: Implementation and Evaluation of Cerebral Microdialysis and Brain Tissue Measurements Supported by UPLC-MS/MS Quantification

Julia Benzel, Gzona Bajraktari-Sylejmani, Philipp Uhl, Abigail Davis, Sreenath Nair, Stefan M. Pfister, Walter E. Haefeli, Johanna Weiss, Jürgen Burhenne, Kristian W. Pajtler and Max Sauter

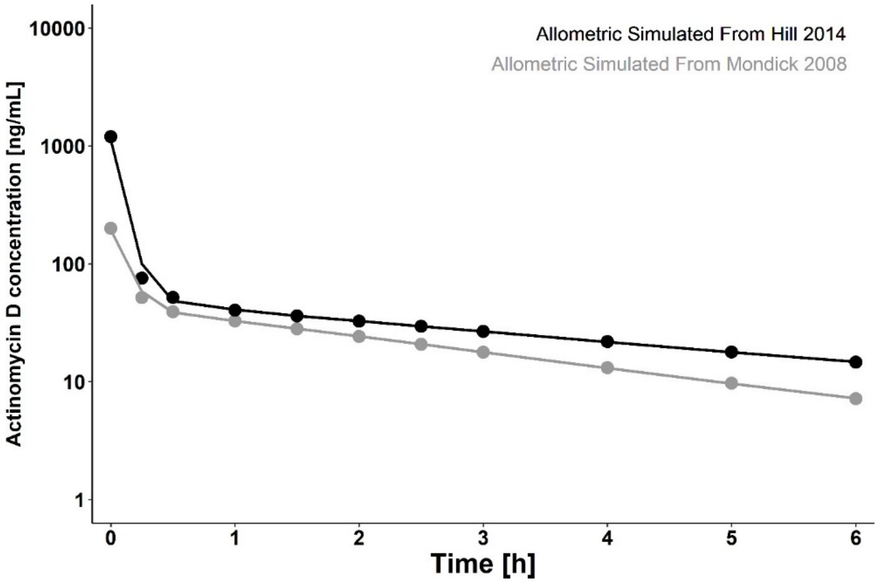

Figure S1. Allometric simulation of actinomycin D´s pharmacokinetic profile in mice according to published data in humans [1,2] by allometrically scaling the clearance and volumes parameters using classic exponents (0.75 for clearances and 1 for volumes) from mouse to human [3].

Table S1. Recovery data.

		Low QC	Medium QC	High QC	IS
		**0.150 ng/mL**	**3.00 ng/mL**	**60.0 ng/mL**	**1.50 ng/mL**
Plasma	Recovery [%]	94.1	91.0	95.0	99.7
	Precision [% CV]	6.43	0.35	1.61	3.58
Brain tissue	Recovery [%]	103.9	104.0	96.6	111.4
	Precision [% CV]	2.92	9.10	8.74	2.07

CV: Coefficient of variation. N = 3 replicates at each QC concentration.

Table S2. Calibration curves of validation batches calculated with linear regression and $1/x^2$ weighting.

Batch	Formula of calibration curve	Coefficient of regression (r^2)
Validation plasma 1	0.0580 x + 0.0014	0.9964
Validation plasma 2	0.0227 x + 0.0015	0.9920
Validation plasma 3	0.0338 x + 0.0008	0.9973
Validation brain tissue 1	0.6332 x + 0.0002	0.9916
Validation brain tissue 2	0.4975 x - 0.0034	0.9904
Validation brain tissue 3	0.4092 x - 0.0091	0.9926

Table S3. Summary of quality control results for plasma actinomycin D.

		Nominal actinomycin D concentrations [ng/mL]			
		**LLOQ**	**Low QC**	**Mid QC**	**High QC**
		**0.050**	**0.150**	**3.00**	**60.0**
		**Within-batch**			
	Mean [ng/mL]	0.047	0.141	3.10	60.2
1	Accuracy [%]	94.0	93.4	103.2	100.3
	Precision [% CV]	13.2	4.95	4.94	3.49
	Mean [ng/mL]	0.045	0.154	3.23	63.5
2	Accuracy [%]	90.0	102.8	107.7	105.8
	Precision [% CV]	1.57	6.98	5.68	3.19
	Mean [ng/mL]	0.050	0.162	3.14	60.4
3	Accuracy [%]	99.0	108.0	104.7	100.7
	Precision [% CV]	8.74	5.79	8.12	6.71
		**Batch-to-batch**			
	Mean [ng/mL]	0.048	0.152	3.16	61.3
	Accuracy [%]	95.9	101.6	105.2	102.2
	Precision [% CV]	8.10	8.17	6.27	5.10

N = 5 replicates at each LLOQ and QC level. CV: coefficient of variation; LLOQ: lower limit of quantification; QC: quality control homogenate.

Table S4. Summary of quality control results for actinomycin D in brain tissue homogenate.

		Nominal actinomycin D concentrations [ng/mL]			
		**LLOQ**	**Low QC**	**Mid QC**	**High QC**
		**0.050**	**0.150**	**3.00**	**60.0**
		**Within-batch**			
	Mean [ng/mL]	0.053	0.169	3.35	57.8
1	Accuracy [%]	106.3	112.7	111.6	96.3
	Precision [%CV]	4.82	1.18	4.10	5.30
	Mean [ng/mL]	0.051	0.164	3.28	56.8
2	Accuracy [%]	101.7	109.2	109.4	94.7
	Precision [%CV]	6.97	3.42	2.27	2.80
	Mean [ng/mL]	0.052	0.158	3.03	53.7
3	Accuracy [%]	104.0	105.6	100.9	89.6
	Precision [%CV]	12.7	2.66	6.68	4.03
		**Batch-to-batch**			
	Mean [ng/mL]	0.050	0.164	3.23	56.4
	Accuracy [%]	99.6	109.4	107.7	94.0
	Precision [% CV]	9.81	3.59	5.96	4.90

N = 5 replicates at each LLOQ and QC level. CV: coefficient of variation; LLOQ: lower limit of quantification; QC: quality control.

Table S5. Summary of quality control results for actinomycin D in micro-dialysate.

	Nominal actinomycin D concentrations [ng/mL]			
	**LLOQ**	**Low QC**	**Mid QC**	**High QC**
	**0.050**	**0.150**	**3.00**	**60.0**
Mean [ng/mL]	0.048	0.162	2.78	52.5
Accuracy [%]	96.0	108.1	92.7	87.5
Precision [% CV]	11.7	3.34	6.12	3.41

N = 5 replicates at each LLOQ and QC level. CV: coefficient of variation; LLOQ: lower limit of quantification; QC: quality control.

Table S6. Summary of quality control results for actinomycin D in cell homogenate.

	Nominal actinomycin D concentrations [ng/mL]			
	**LLOQ**	**Low QC**	**Mid QC**	**High QC**
	**0.050**	**0.150**	**3.00**	**60.0**
Mean [ng/mL]	0.050	0.156	3.20	66.4
Accuracy [%]	100.3	103.9	106.7	110.6
Precision [% CV]	12.2	6.21	7.51	3.75

N = 5 replicates at each LLOQ and QC level. CV: coefficient of variation; LLOQ: lower limit of quantification; QC: quality control.

Table S7. Stability data of the plasma validation.

	**Low QC** 0.150 ng/mL	**Medium QC** 3.00 ng/mL	**High QC** 60.0 ng/mL
Freeze-and-thaw stability			
Accuracy [%]	93.6	92.0	87.6
Precision [% CV]	5.80	7.59	2.65
	1-month plasma stability at -25 °C		
Accuracy [%]	95.6	98.3	100.7
Precision [% CV]	14.7	2.44	2.00
2 days buffer stability at RT			
Accuracy [%]	108.7	113.5	104.7
Precision [% CV]	5.21	1.69	8.49
	6-month stability in solution at 4°C		
Accuracy [%]	88.3	86.4	87.2
Precision [% CV]	1.60	5.54	3.34

CV: Coefficient of variation. N = 3 replicates at each QC concentration. RT: room temperature.

Table S8. Results of the incurred sample reanalysis.

**Mouse #**	**Time of sampling [h]**	**Actinomycin D concentration [ng/mL] Original analysis**	**Actinomycin D concentration [ng/mL] Incurred reanalysis**	**Deviation from mean [%]**
5	22	1.40	1.19	16.2
6	6	1.31	1.17	11.3
6	22	1.36	1.16	15.9
7	6	5.51	5.23	5.21
7	22	4.48	5.17	14.3

Table S9. Summary of quality control results for actinomycin D quantification of minimally diluted QC samples with calibration samples spiked with regular solutions.

	Nominal actinomycin D concentrations [ng/mL]		
	**Low QC**	**Mid QC**	**High QC**
	**0.150**	**3.00**	**60.0**
Mean [ng/mL]	0.142	2.80	53.8
Accuracy [%]	94.7	93.5	89.8
Precision [% CV]	10.0	9.03	1.43

N = 3 replicates at each LLOQ and QC level. CV: coefficient of variation; QC: quality control.

Table S10. Matrix effect data.

		Low QC	Medium QC	High QC	IS
		0.150 ng/mL	3.00 ng/mL	60.0 ng/mL	1.50 ng/mL
Plasma	Matrix effect [%]	66.2	74.2	73.1	66.2
	Matrix effect IS-normalized [%]	104.6	109.8	102.0	
	Precision [% CV]	13.7	1.05	6.91	1.08
Brain tissue	Matrix effect [%]	56.0	63.2	62.3	52.0
	Matrix effect IS-normalized [%]	110.3	114.5	112.8	
	Precision [% CV]	8.04	2.75	4.89	2.46

CV: Coefficient of variation. N = 3 replicates at each QC concentration.

Table S11. *In vitro* probe performance comparison.

Probe	CAM7 (Cat.No.:8012411) n = 2	CMA8 (Cat.No.:8012201) n = 1	MD-2211 n = 5
Material	Polyethersulfone (PES)	Polyacrylethersulfone (PAES)	Cellulose
MWCO	55kDa	20kDa	35kDa
RD recovery rate (%)	81.6 ± 9.9	88.83	79.4 ± 2.8

RD: Retrodialysis.

Table S12. Actinomycin D in vitro retrodialysis results.

	Probe 1	Probe 2	Probe 3	Probe 4	Mean
1 h (ng/mL)	11.2	20.0	3.82	5.37	
2 h (ng/mL)	9.78	13.6	7.54	16.9	
3 h (ng/mL)	16.4	17.0	9.84	13.3	
Stock (ng/mL)†	95.6	73.6	67.5	76.6	
Recovery rate (%)	87.0	77.1	89.5	84.5	84.5 ± 5.4

† Expected concentration of 100 ng/mL.

Table S13. Actinomycin C in vitro retrodialysis results using 1 % BSA.

	Probe 1	Probe 2	Mean
1 h (ng/mL)	6.27	4.14	
2 h (ng/mL)	6.38	9.59	
3 h (ng/mL)	11.8	13.6	
Stock (ng/mL)†	67.4	71.08	
Recovery rate (%)	87.9	87.2	87.6 ± 0.5

† Expected concentration of 100 ng/mM.

Table S14. Summary of quality control results for actinomycin C in micro-dialysate.

	Nominal actinomycin C concentrations [ng/mL]			
	LLOQ 0.100	Low QC 0.300	Mid QC 6.00	High QC 75.0
Mean [ng/mL]	0.083	0.313	6.67	80.1
Accuracy [%]	83.2	104.3	111.2	106.8
Precision [% CV]	3.65	5.78	4.95	11.09

N = 5 replicates at each LLOQ and QC level. CV: coefficient of variation; LLOQ: lower limit of quantification; QC: quality control.

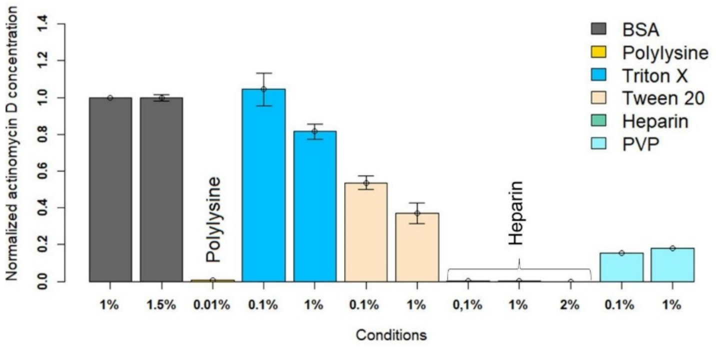

Figure S2. Coating strategies to avoid non-specific binding of actinomycin D in case of BSA and Tritonx100. Microdialysis tubes were coated for 1 h with indicated chemicals and drug concentrations normalized to the nominal value. Data is expressed as mean ± standard deviation. Coatings were not stable for the whole duration of microdialysis procedure (data not shown).

References

1. Mondick, J.T.; Gibiansky, L.; Gastonguay, M.R.; Skolnik, J.M.; Cole, M.; Veal, G.J.; Boddy, A.V.; Adamson, P.C.; Barrett, J.S. Population pharmacokinetic investigation of actinomycin-D in children and young adults. *The Journal of Clinical Pharmacology* **2008**, *48*, 35–42.
2. Hill, C.R.; Cole, M.; Errington, J.; Malik, G.; Boddy, A.V.; Veal, G.J. Characterisation of the clinical pharmacokinetics of actinomycin D and the influence of ABCB1 pharmacogenetic variation on actinomycin D disposition in children with cancer. *Clinical pharmacokinetics* **2014**, *53*, 741–751.
3. Boxenbaum, H. Interspecies pharmacokinetic scaling and the evolutionary- comparative paradigm. **1984**, *15*, 1071–1121.
